# The Role of Neuropeptide Y in the Pathogenesis of Alzheimer’s Disease: Diagnostic Significance and Neuroprotective Functions

**DOI:** 10.3390/neurolint16060100

**Published:** 2024-11-01

**Authors:** Ksenia Shapovalova, Yana Zorkina, Olga Abramova, Alisa Andryushchenko, Vladimir Chekhonin, Georgy Kostyuk

**Affiliations:** 1 Mental-Health Clinic No. 1 Named After N.A. Alekseev, Zagorodnoe Highway 2, 115191 Moscow, Russia; shapovalova_ksyusha@bk.ru (K.S.); abramova1128@gmail.com (O.A.); alissia.va@mail.ru (A.A.); kgp@yandex.ru (G.K.); 2 Department of Basic and Applied Neurobiology, V. Serbsky Federal Medical Research Centre of Psychiatry and Narcology, Kropotkinsky Per. 23, 119034 Moscow, Russia; chekhoninnew@yandex.ru; 3 Department of Medical Nanobiotechnology, Pirogov Russian National Research Medical University, 117997 Moscow, Russia; 4 Department of Psychiatry, Federal State Budgetary Educational Institution of Higher Education “Moscow State University of Food Production”, Volokolamskoye Highway 11, 125080 Moscow, Russia; 5 Department of Mental Health, Faculty of Psychology, M. V. Lomonosov Moscow State University, 119991 Moscow, Russia; 6 Department of Psychiatry and Psychosomatics, I. M. Sechenov First Moscow State Medical University (Sechenov University), 119435 Moscow, Russia

**Keywords:** neuropeptide Y, Alzheimer’s disease, CSF, blood plasma, transgenic animal, neuroprotection

## Abstract

**Background**. Alzheimer’s disease (AD) is one of the most common neurodegenerative diseases. It has been suggested that the factors that cause pathologic changes and lead to the development of AD may also include changes in certain neuropeptides. The implication of the neuropeptide (NPY) in the pathogenesis of AD and its potential therapeutic role is possible due to the following properties: involvement in adult neurogenesis, regulatory effects on the immune system, the inhibition of potential-dependent Ca^2+^ channels, and the reduction in glutamate excitotoxicity. The aim of our review was to summarize recent data on the role of NPY in AD development and to explore its potential as a biomarker and a possible therapeutic target. **Materials and methods**. We performed a systematic review of studies, for which we search using the keywords “Alzheimer’s disease and neuropeptide Y”, “Alzheimer’s disease and NPY”, “AD and NPY”, “Neuropeptide Y and Neurodegenerative disease”. Nineteen articles were included in the review. **Results**. The NPY levels in cerebrospinal fluid and plasma have been found to be reduced or unchanged in AD patients; however, these findings need to be confirmed in more recent studies. Data obtained in transgenic animal models support the role of NPY in AD pathogenesis. The neuroprotective effects of NPY have been demonstrated in vitro and in vivo in AD models. **Conclusion**. The findings may open new possibilities for using NPY as a diagnostic marker to detect AD at earlier stages of the disease or as a potential therapeutic target due to its neuroprotective properties.

## 1. Introduction

Alzheimer’s disease (AD) is one of the most common neurodegenerative diseases. AD contributes to 60–70% of all cases of dementia [1]. In recent decades, the number of patients with cognitive impairment has only been increasing. In 2015, the global incidence was estimated at 50 million people [2]. Today, dementia is the seventh leading cause of death and one of the leading causes of disability in the elderly, with women being more affected than men [1]. AD is a severe disease, the first symptoms of which include memory loss for recent events and forgetfulness. As the disease progresses, it is followed by loss of memory for distant past events, speech disorders, cognitive decline, and loss of self-care [3,4]. Among the theories regarding the pathophysiologic mechanisms of AD development there are the amyloid hypothesis and tau-hypothesis [5,6]. The amyloid hypothesis is that Aβ plaques form and are deposited in different regions of the brain. These plaques are recognized by the brain as foreign material, which triggers an inflammatory and immune response by activating microglia and releasing cytokines that ultimately lead to cell death and neurodegeneration [5,7]. The tau hypothesis is associated with the dysfunction of neuronal tau proteins. Tau proteins have a binding domain that is involved in the polymerization and stabilization of microtubule assembly to maintain the integrity of the nerve cell cytoskeleton. This binding is regulated by the phosphorylation of serine/threonine residues via various kinases, including cyclin-dependent kinase-5 (CDK5). The disruption of CDK5 regulation leads to the hyperphosphorylation of tau protein, which reduces the affinity of tau proteins for microtubules. Hyperphosphorylated tau protein forms neurofibrillary tubules and is deposited in the cytosol, resulting in the impairment of its primary function. This affects normal neuronal functions such as synaptic transmission, axonal transport, and signaling, and the cell gradually degenerates [6,7]. Thus, Aβ and tau protein form aggregates and cause synaptic dysfunction, impaired synaptic plasticity, and neuronal death, which contribute to neurodegeneration and dementia [7]. Additionally, hypotheses related to oxidative stress, in particular, the peroxidation of membrane lipids and its impact on cholinergic transmission and receptor–protein coupling, have been proposed [8]. Several factors, including inflammation, genetic factors, and viruses, are believed to contribute to the pathogenesis of the disease [9]. Neuroinflammation plays a central role in the pathogenesis of AD. Acute inflammation plays a protective role by preventing brain damage, including from plaques, but the constant activation of microglia renders it unable to remove plaques, but it retains its ability to produce pro-inflammatory cytokines. This results in an imbalance between the pro-inflammatory and anti-inflammatory cytokines. These cytokines activate cyclin-dependent kinases, leading to the hyperphosphorylation of tau protein and increased Aβ plaque formation [7,10]. Additionally, other glial cell astrocytes may contribute to neurodegeneration and inflammation by recruiting peripheral inflammatory cells, the activation of microglia, and their own neurotoxic activity [11]. It has been suggested that factors that cause pathologic changes and lead to the development of AD can also affect some neuropeptides. One example is the decrease in neuropeptide Y (NPY) found in postmortem brain studies in AD patients [12].

NPY is a 36-amino acid neuropeptide that is widely expressed in the central and peripheral nervous system of humans and other mammals [13]. NPY protein is mainly produced and secreted by GABAergic interneurons in the brain during prolonged neuronal activity. However, it can also be present in astrocytes and some projection neurons. In addition, it can enter the brain from the bloodstream by crossing the blood–brain barrier [14].

The functions of NPY include the regulation of the central nervous system (CNS), participation in the inflammatory response of the CNS, influence on eating behavior and the cardiovascular system, and the regulation of heart rate [15,16,17]. The excessive expression of NPY is considered as one of the possible causes of hypothalamic obesity. NPY, acting on the hypothalamic satiety and hunger center, increases food intake and induces hunger [18]. NPY receptors are involved in various gastrointestinal functions including intestinal motility, electrolyte balance, and nutrient and water absorption [19]. The role of NPY in the development of depression, posttraumatic stress disorder, and chronic pain syndrome has been widely studied [20,21]. The involvement of NPY in the pathogenesis of depression is confirmed by the differential expression of the neuropeptide in various animal models of depression [22]. Along with that, increased NPY levels have been found in the cerebrospinal fluid (CSF) of patients with severe depression [23].

## 2. NPY Receptors

NPY realizes its biological functions by binding to and activating receptors in various regions of the brain. NPY receptors are G-protein-coupled receptors and include Y1, Y2, Y4, Y5, and Y6 subtypes [24]. The physiological role of the Y6 receptor has not yet been fully studied in some mammals, including humans. Y1 and Y2 receptors are the most abundant NPY receptors. Y1, Y2, and Y5 receptors are responsible for the regulation of eating behavior in animals; Y1, Y2, and Y4 are involved in the regulation of anxiety and depression in animals. NPY also affects cell migration, cytokine release, and antibody production through Y1 receptors [25,26,27].

**Figure 1:**
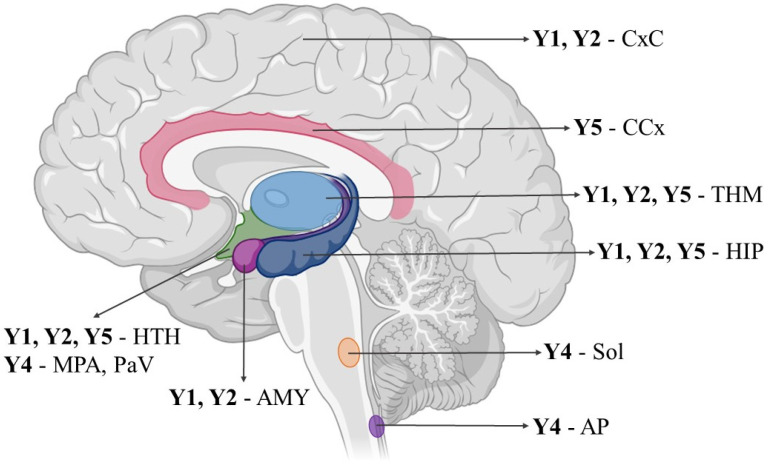
NPY receptors in brain structures. AMY—amygdala; AP—area postrema; CCx—cingulate cortex; CxC—cortex cerebri; HIP—hippocampus; HTH—hypothalamus; MPA—medial preoptic area; PaV—paraventricular nucleus; sol—solitary nucleus; THM—thalamus. The scientific image and illustration software BioRender.com was used to create the illustration.

In the CNS, NPY receptors are located on neurons (Y1, Y2), astrocytes (Y1), nerve stem cells (Y1), microglia (Y1, Y2, Y5), and endothelial cells that form the blood–brain barrier (Y1, Y2) [15,28,29,30]. NPY is expressed in multiple brain regions, but the main sources of NPY are the hypothalamic arcuate nucleus, the locus coeruleus, nucleus tractus solitarii, and the septohippocampal nucleus [31]. NPY can be found predominantly in the cerebral cortex, the limbic system, thalamic regions, and certain regions of the medulla oblongata [32] (Figure 1).

This wide distribution of different NPY receptor subtypes in different brain regions explains the diverse functions that NPY participates in regulating through its receptors. For example, it has been shown that NPY via Y1, Y2, and Y5 receptors can regulate neurotransmission in multiple circuits of the limbic and autonomic nervous system [33]. NPY can directly stimulate NO release via Y1 and Y2 receptors in the neurons of the tuberal and some other hypothalamic regions, and through this pathway may participate in the regulation of blood flow and other systems [33]. Moreover, Y1 receptors in the nucleus accumbens are involved in both the post- and pre-synaptic effects of NPY and influence certain neurons associated with movement control. In addition, NPY is involved in the characteristic functions of the nucleus accumbens via Y1, such as in alcohol consumption, drug dependence, food intake, anxiety, and depression [34]. The vasopressin and oxytocin neurons of the hypothalamic–pituitary tract also appear to express NPY receptors such as Y5 and possibly Y1, likely accounting for the role of NPY in the regulation of circadian rhythms [33]. Some pathological states are regulated through NPY receptors in specific areas of the brain. The Y1 receptor located in the forebrain and hippocampus and the Y2 receptor in the cortex and amygdala are involved in the regulation of anxiety behavior [31]. Changes in Y1 and Y2 receptor expression were observed in the hippocampus of patients with temporal lobe epilepsy [35].

Studies in humans and rodents have shown that NPY levels in the brain are altered in several neurodegenerative diseases, including AD. There is a decrease in NPY in the hippocampus and cortex in AD patients, which is likely the result of the extensive neurodegeneration that occurs in these brain regions as the disease progresses [36]. The NPY system has therapeutic potential in the most common neurodegenerative diseases because it can attenuate the pathologic mechanisms that lead to neurodegeneration [36]. The neuroprotective properties of NPY are due to its receptors, which participate in different neuroprotective mechanisms. NPY in vitro protects the hippocampal, cerebral cortex, and retinal cells from glutamate excitotoxicity by activating Y2- and Y5-receptors. NPY can also attenuate oxidative stress by inhibiting reactive oxygen species through the modulation of Y1 and Y2 receptors [37]. In an animal experiment, the Y1 receptor was shown to play an important role in AD-related cognitive impairment, as memory impairment in rats was improved by the NPY and Y1 receptor agonist, while the Y1 receptor antagonist had the opposite effect [31]. The neuroprotective properties of NPY may be mediated through Y2 receptors and may include the activation of mitogen-activated protein kinase and Akt signaling pathways [38].

## 3. Potential Role of NPY in AD

The implication of NPY in the pathogenesis of Alzheimer’s disease and its potential therapeutic role is based on several properties of the peptide:

1. Involvement in neurogenesis, including adult neurogenesis;
2. Suppression of microglia activation and regulatory effects on the immune system;
3. Inhibition of intracellular Ca^2+^ concentration and protection mitochondria from reactive oxygen species (ROS);
4. Autophagy stimulation.

The main symptom of AD is memory impairment. The hippocampus is the region of the brain responsible for memory formation and the conversion of short-term memory into long-term memory. Neurogenesis in adulthood occurs in the dentate gyrus of the hippocampus. Neurogenesis declines during aging [39]. The number of dablcortin-positive cells (a microtubule-associated protein expressed almost exclusively in immature neurons) and stem cells is reduced in Alzheimer’s patients compared to healthy individuals [40]. The authors proposed a correlation between cognitive function and neurogenesis in AD patients. NPY receptors are located on neural stem cells (nestin- and doublecortin-positive cells), and are involved in the proliferation, differentiation, migration, and functional integration of newly formed neurons [29]. NPY is involved in the modulation of adult neurogenesis in the dentate gyrus of the hippocampus, also affecting migration, proliferation, and differentiation [29]. Furthermore, NPY mediates intercellular interactions between different cell types in the central nervous system [29]. In addition, NPY promotes axon sprouting and neuronal differentiation through the activation of the SAPK/JNK pathway (stress-activated protein kinase/c-Jun N-terminal kinase) [41]. Through Y1 receptors, NPY stimulates astrocyte proliferation; through Y2 and Y5 receptors on endothelial cells, it stimulates angiogenesis, which can also enhance neurogenesis [29]. Brain-derived neurotrophic factor (BDNF) plays a crucial role in neuronal survival, growth, differentiation, and neurogenesis. BDNF concentration is decreased in the blood and brain of patients with Alzheimer’s disease [38]. It has been shown that NPY and BDNF can stimulate each other’s expression. NPY altered the expression of microRNAs involved in BDNF expression [42]. NPY positively regulates BDNF expression in rat cortical neurons exposed to Aβ42 [43]. NPY has also shown the effect of NPY on hippocampus-dependent learning and memory in animal models via the activation of Y1 and Y2 receptors [44].

Another hypothesis of AD is related to microglia activation and neuroinflammation. Microglia are resistant macrophages of the CNS and a component of innate immunity. Microglia proliferation and activation occurs around amyloid plaques in the brain of humans with AD [45]. This has a protective effect, as amyloid fibrils become more densely packed and potentially less toxic, preventing further accumulation of Aβ on existing plaques. However, pathologic microglia activation can induce neuronal damage and synaptic loss. Microglia also produces pro-inflammatory cytokines that cause neuroinflammation, leading to neuronal damage [45]. NPY inhibits microglia activation and interleukin 1 beta release by interacting with the Y1 receptor [29]. NPY receptors are located on different types of immune cells (monocytes, macrophages, dendritic cells, natural killer cells, lymphocytes, granulocytes), and can directly affect their function. Through Y1 receptors, it inhibits the activation and regulation of proliferation, differentiation, and cytokine secretion; through Y1/Y2/Y5, it also mediates phagocytosis and migration [46]. Thus, NPY has a direct impact on neuroinflammatory processes.

Aβ can bind to the proteins and lipids of cell membranes and increase their permeability. Increased cell membrane permeability leads to an influx of Ca^2+^. Increased cytoplasmic Ca^2+^ leads to toxic cellular damage as observed in AD pathology [47]. There are several mechanisms by which Aβ can disrupt membrane integrity and increase intracellular Ca^2+^ concentration. First, Aβ can induce the hyperactivation of NMDA receptors, leading to the increased production of ROS and causing mitochondrial dysfunction. Together, these processes are known as glutamate excitotoxicity [48]. Second, Aβ oligomers are able to incorporate into cell membranes and form large single ion channel pores. Third, voltage-gated calcium channels (VGCCs) are affected in AD as shown in in vitro and in vivo models [48]. NPY affects the reduction in intracellular Ca^2+^ concentration through all the described mechanisms. NPY can reduce glutamate excitotoxicity, inhibit glutamate receptor overactivity, and protect hippocampal and cortical cells from necrosis or apoptosis through the activation of Y2 and Y5 receptors. This neuroprotective effect is mediated by the activation of protein kinase A and p38K, key proteins in various intracellular signaling pathways [49]. NPY may exhibit neuroprotective effects against glutamate-induced excitotoxicity in many brain regions such as the hippocampus and striatum [45,46,47]. NPY can reduce oxidative stress by inhibiting ROS through the modulation of Y1 and Y2 receptors [50]. NPY protects mitochondria from oxidative damage by suppressing nitric oxide production [51]. NPY impacts the ability of the Aβ to form Ca^2+^ permeable pores in neuronal membranes [52] and can inhibit VGCCs [53].

The key point in the development of AD is the accumulation of pathologically folded proteins, such as Aβ and tau protein. Normally, when pathologic misfolded proteins appear in a cell, they are degraded by autophagy; thus, the cell restores homeostasis. Unlike most other cells, neurons are extremely vulnerable to autophagy disruption because they are not actively dividing cells [54]. When autophagy pathways are disrupted, toxic misfolded proteins accumulate and become visible as intracellular deposits, leading to the disruption of cell activity and then to cell death. In 2015, it was shown for the first time that NPY stimulates autophagy in hypothalamic neurons through the activation of Y1 and Y5 receptors [55]. This has been shown in hypothalamic neuronal cell culture and in mice with NPY overexpression in the hypothalamus. This brain region is crucial for the development of whole-body aging and has an impact on lifespan. In addition, authors showed that PI3K, ERK, and PKA signaling pathways are involved in these processes [56].

To investigate the role of NPY in the pathogenesis of AD, some studies have included the application of new omics technologies, consisting of the analysis of completely molecular profiles (e.g., genomic, transcriptomic, proteomic, etc.). These studies have also shown some results. The single-cell transcriptomics method in conjunction with the established abca7 gene knockout model (a known AD risk gene) in danio fish has shown abca7-dependent interconnection between neurons and glial cells through NPY signaling, which is essential for synaptic integrity, and whose disruption is a risk factor for AD due to decreased brain stability [43]. Genome-wide association studies that have been conducted on large samples of AD patients have found some polymorphisms of the NPY gene (rs73084930, rs62450407) associated with cognitive function in AD [57,58].

**Figure 2:**
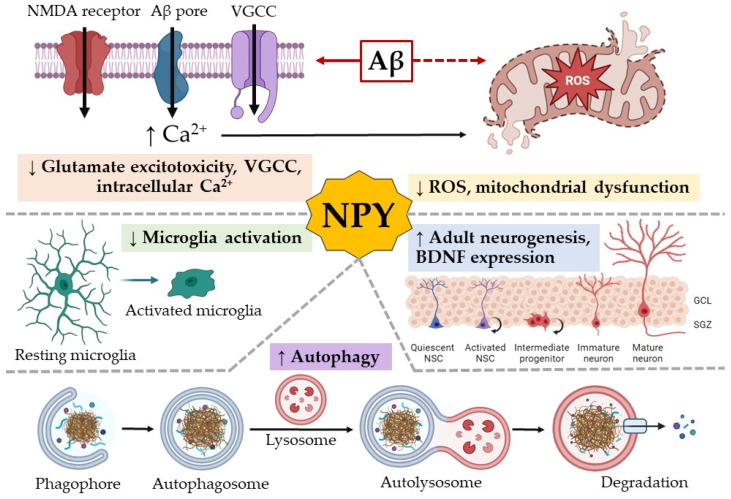
NPY and its action on pathogenetic pathways of AD. NPY acts on different pathways of AD pathogenesis. 1. Aβ can induce hyperactivation of NMDA receptors and voltage-gated calcium channels (VGCCs), Aβ oligomers are able to incorporate into cell membranes and form large single-ion channel pores. All of these lead to an increase in intracellular Ca^2+^ concentration. Increased levels of intracellular Ca^2+^ can damage mitochondria. NPY reduces intracellular Ca^2+^ concentration by inhibiting VGCC, reducing NMDA receptor excitotoxicity and affecting the ability of the Aβ to form Ca^2+^ permeable pores in neuronal membranes. NPY can reduce reactive oxygen species (ROS) and protect mitochondria. 2. NPY reduces microglia activation and neuroinflammation. 3. NPY stimulates neurogenesis, including in adulthood. NPY and BDNF can stimulate each other’s expression, which also enhances neurogenesis. 4. NPY stimulates autophagy in hypothalamic neurons, which may protect the cell from Aβ toxicity. NSC—neural stem cells; GCL—granule cell layer; SGZ—subgranular zone. The scientific image and illustration software BioRender.com was used to create the illustration.

The effects of NPY on the AD pathogenetic pathways are shown in Figure 2.

Thus, NPY is a regulator of multiple processes and is involved in key pathways of AD pathogenesis. The aim of our study was to summarize recent data on the role of NPY in the AD pathogenesis, as well as to investigate it as a potential biomarker of AD development and a possible target in the therapy of the disease.

## 4. Materials and Methods

We performed a systematic review of studies, for which we reviewed articles using PubMed and Google Scholar search using the keywords “Alzheimer’s disease and neuropeptide Y”, “Alzheimer’s disease and NPY”, “AD and NPY”, “Neuropeptide Y and Neurodegenerative disease”, “Neuropeptide Y”, “Alzheimer’s disease”, and “neuropeptide Y”. The search yielded 137 articles. Articles not relevant to the topic of our review were excluded. As a result of the selection process, 19 articles were included in the review.

## 5. Results

### 5.1. NPY as Diagnostic Marker in CSF

A large number of studies have focused on the NPY levels in CSF, reflecting the potential of NPY to become a diagnostic biomarker for AD. In a study by Minthon et al. [59], NPY concentration in CSF was measured in patients with frontal temporal dementia (FTD) and AD. The findings revealed that NPY was significantly reduced in patients suffering from dementia of the Alzheimer’s type, with lower concentration observed in more severe stages of the disease. The results on decreased NPY levels in AD patients were further confirmed by the study conducted by Alom et al. [60]. Edvinsson et al. [61] performed similar research [59], examining patients with FTD and AD, but their findings differed from those of the previous study. The NPY level in both groups of patients was not statistically significantly different from the control group. At the same time, a significant correlation with the duration of neurodegenerative disease was observed in AD patients. The patients experiencing longer disease durations had lower NPY levels in CSF. Despite these findings, other studies have not confirmed the reduction in NPY levels in CSF [62,63]. These inconsistent results may be, in part, explained by the method of NPY analysis, as the intra- or extra-cellular processing of NPY may or may not result in NPY being detected in the assay [36]. It may also be taken into account that the degree of change in the NPY levels observed in brain tissue, including neuronal damage, may influence changes in the CSF. It may take a certain high number of damaged neurons expressing NPY for this to translate into a decrease in its levels in the CSF [36,64].

### 5.2. NPY as Diagnostic Marker in Blood Plasma

Contradictory data have been found in studies on NPY levels in human plasma. Some studies have shown a significant decrease in the plasma concentration of the peptide [65], while other studies have not confirmed these findings [66]. This discrepancy in the obtained data are difficult to explain, and may be due to the fact that earlier studies used less sensitive measurement methods. Other problems in the discrepancy could potentially lie in issues such as insufficient or inappropriate experimental groups or differences in methods of obtaining biomaterial. Given that the results of only two studies with really small numbers of participants (up to 25 people per group) are currently available, it is too early to draw definitive conclusions about NPY blood levels in AD. A limitation of our study is the lack of new articles investigating NPY in plasma and CSF. The data on NPY content in CSF and plasma of AD patients are presented in Table 1.

### 5.3. Studies in Transgenic Animals

A number of studies were conducted on transgenic animal models of AD.

Mahar I. et al. [67] studied interneuronal changes in the hippocampus of 1-month-old TgCRND8 mice with AD at the early (pre-symptomatic) stages of the disease, before the accumulation of Aβ. The findings indicated that the number of neurons expressing NPY and parvalbumin was reduced in some subregions of the hippocampus. A more detailed analysis revealed that the total number of NPY-expressing neurons was reduced in hippocampal subregions CA1/2 and dentate gyrus, but not in CA3 or the subiculum. Based on the results of the study, the authors concluded that early functional changes in the hippocampal interneurons occurred before amyloid deposition at the age of 1 month, leading to cognitive dysfunction in AD.

In the Palop 2007 study conducted on hAPP FAD transgenic mice characterized by high seizure activity, alterations in NPY expression levels in the hippocampus were observed. NPY receptors affected glutamate release, which contributed to increased seizure activity in mice [68].

Another study [69] confirming the earlier findings, found that NPY-expressing GABAergic neurons, unlike other GABAergic neurons in the hippocampus, were the most vulnerable to Aβ accumulation. Their numbers were reduced in the CA1-CA2 (pyramidal, stratum oriens, stratum radiatum, and molecular layers), CA3 (specifically in stratum oriens), and dentate gyrus (specifically in the polymorphic layer) in TgCRND8 mice compared to nontransgenic controls. Taken together, the data obtained in this study provides new evidence for the significance of disruptions in the hippocampal GABAergic neuronal network as a mechanism contributing to AD-related consequences, such as abnormal neuronal excitability. Different hippocampal GABAergic neuron types show surprisingly different regional and subregional vulnerability to Aβ accumulation.

Ramos B. et al. [70] studied the NPY interneuron population in the hippocampus of 6-month-old PS1 × APP transgenic mice with AD. They revealed a significant decrease in NPY mRNA expression in PS1 × APP mice as early as 4–6 months of age, which progressed to lower values during aging. The number of NPY immunolabelled interneurons was substantially reduced in PS1 × APP mice compared to the control groups. These results confirm the early and extensive degeneration of dendritic inhibitory interneurons. Thus, NPY mRNA expression can be considered as specific and early molecular markers of AD in the PS1 × APP model. These neuropeptides may also be beneficial as molecular tools for evaluating the efficacy of potential AD therapies in this mouse model.

Krezymon A. et al. [71] studied the association of the AD stage with the expression of markers for hippocampal dysfunction, as well as NPY expression levels, depending on the age and severity of the disease in Tg2576 mice. This study found that, as mice age, a complex pattern of changes develops in the dentate gyrus, including aberrant NPY expression. This reflects a profound remodeling of inhibitory and excitatory circuits in the dentate gyrus. Aberrant NPY expression was not observed in the molecular layer of the dentate gyrus in Tg2576 mice under 18 months of age. Thus, the sprouting of NPY fibers observed in the aged Tg2576 mice likely enhanced granule cell inhibition, which in turn may have been responsible for the cognitive impairment in these mice. Taken together, these data suggest that the abnormal expression pattern of NPY in the hippocampus of Tg2576 mice may reflect a compensatory inhibitory response to Aβ-induced aberrant excitatory neuronal activity.

Ishii M. et al. [72] demonstrated that 3-month-old Tg2576 mice exhibited weight loss with a marked reduction in fat mass, low plasma leptin levels, and increased energy expenditure without changes in eating behavior prior to amyloid plaque formation. Orexigenic NPY expression in the hypothalamus in response to low leptin levels was abnormal under basal and fasting conditions. In addition, the arcuate NPY neurons exhibited abnormal electrophysiological responses to leptin in the hypothalamic brain slices from the Tg2576 mice or beta-amyloid-treated wild-type mice. These results suggest that excessive Aβ may potentially impair hypothalamic arcuate NPY neurons, leading to weight loss and pathologically low leptin levels in the early stages of the disease, which progressively worsen as amyloid burden increases.

Overall, the findings from these studies indicate that changes in NPY occur in transgenic animals, both at early stages (subregional decreased number of immunoreactive NPY and PV cells in 1-month-old TgCRND8 mice with AD, increased vulnerability of NPY-expressing GABAergic neurons in the same model, decreased NPY mRNA expression in PS1 × APP mice at 4–6 months), and as the disease ages and progresses (aberrant NPY expression, reflecting profound remodeling of inhibitory and excitatory circuits in the dentate gyrus in Tg2576 mice). Thus, studies on transgenic models of AD suggest that changes in NPY are involved in almost all the stages of AD pathogenesis and are a key factors in disease development.

### 5.4. Neuroprotective Properties of NPY

The neuroprotective effects of NPY have been studied in a number of in vitro and in vivo studies. In vitro studies have demonstrated a reduction in Aβ toxicity when cells are pretreated with NPY. Rose et al. [73] found that pre-incubation with amidated C-terminal fragments of NPY derived from neprilysin protects primary human cortical neurons from Aβ toxicity. The same group showed that the intravenous administration of these fragments prevents neuronal degeneration in APP transgenic mice.

Another study [74] evaluated the effect of a single intracerebroventricular injection of NPY 15 min before the intravenous administration of aggregated peptide Aβ 1–40 on behavioral and neurochemical parameters associated with oxidative stress in mice. The findings indicated that the central administration of NPY prevents Aβ-induced depressive-like behavior and spatial memory deficits in mice. This response was mediated, at least in part, by the activation of Y2 receptors.

Croce et al. [75] studied the neuroprotective properties of NPY on an SH-SY5Y neuroblastoma cell line treated with Aβ. They found that NPY increased the survival of SH-SY5Y cells, counteracted Aβ toxicity, and restored neurotrophin levels in these cells. In another study, Croce et al. [76] investigated the neuroprotective effect of NPY on cortical neurons. Pretreatment with NPY counteracted the toxic effect of Aβ by increasing cell viability. Moreover, NPY increased intracellular synthesis and normalized nerve growth factor (NGF) release, and induced a decrease in mRNA expression. Croce et al. [42] also demonstrated that NPY pretreatment decreased miR-30a-5p expression and increased brain-derived neurotrophic factor (BDNF) mRNA and protein expression 24 and 48 h after incubation with Aβ. This suggests that NPY modulates BDNF and its regulatory microRNA miR-30a-5p in the opposite direction, potentially contributing to the neuroprotective effect of NPY in Aβ-exposed cortical neurons.

Spencer et al. [77] investigated neuroprotective properties in vitro and in vivo by administering lentiviruses expressing NPY with an apoB tagret. These vectors increased the survival of B103 rat neuroblastoma cells or adult rat hippocampal progenitor cells treated with Aβ. In transgenic mice, a single intraperitoneal injection increased neurogenesis and synaptic density, and decreased astrogliosis, and also improved spatial memory and locomotor activity in mice. These data indicate the ability of NPY to repress neuroinflammatory responses and neurodegeneration.

Thus, studies suggest that NPY administration in AD models produces positive effects on behavior and cognitive function in animals. NPY counteracts Aβ toxicity through multiple mechanisms, including the activation of Y2 receptors, the prevention of oxidative stress, the modulation of BDNF and its regulatory microRNA miR-30a-5p, as well as by increasing NGF production. These findings support the neuroprotective and therapeutic potential of NPY in AD treatment.

The total data are summarized in Table 2.

## 6. Conclusions

NPY and its receptors are widely expressed in the brain, and their expression levels are altered in some pathological conditions. The role of NPY in the development of AD has been demonstrated in a number of studies. The NPY levels in the CSF and plasma of AD patients have been found to be reduced or unchanged, although these findings need to be confirmed in more recent research. The data from transgenic animal models suggest the role of NPY in the pathogenesis of AD. Taken together, the results of the described studies support the idea that changes in NPY are common in AD and may contribute to its development.

On the other hand, the NPY acts through several mechanisms to exert a neuroprotective effect (stimulation of neuroproliferation and neuronal survival, activation of autophagy for the clearance of aggregate proteins, suppression of excitotoxicity and attenuation of neuroinflammation), so it has therapeutic potential in neurodegenerative diseases, since it can attenuate the pathological mechanisms that lead to neurodegeneration. However, it is necessary to take into account some limitations for its use, namely poor penetration through the blood–brain barrier, low bioavailability in oral administration, and short half-life [37]. However, animal studies have demonstrated the possibility of using an intranasal form of the NPY, as it has been shown to be able to penetrate into the brain via the olfactory and trigeminal nerves in the nasal cavity [78].

Nevertheless, the results of neuropeptide studies have not yet reached clinical application, so further studies are needed to fully understand the complex role of NPY in preventing or halting the progression of neurodegenerative diseases, as well as the safety of its use in the clinic.

## Figures and Tables

**Table 1:** NPY levels in CSF and plasma in AD patients. CTL—control; AD—Alzheimer’s disease; VAD—vascular dementia.

Material	Sample	NPY Level	NPY Levels in AD	NPY Levels in Normal	Correlation of NPY Level with AD Stage	Reference
CSF	CTL—11; FTD—11; AD type dementia—17	Decreased compared to CTL	109 + 21 pmol/L	139 ± 12 pmol/L	+	Minthon, 1990 [59]
CSF	CTL—19; AD—20	Decreased compared to CTL	69.5 ± 36.7 pg/mL	103 ± 21.8 pg/mL	+	Alom, 1990 [60]
CSF	CTL—11; FTD—41; AD type dementia—39	No difference	120 ± 29 pmol/L	139 ± 11 pmol/L	+	Edvinsson, 1993 [61]
CSF	CTL—26; AD—27; AD with extrapyramidal symptoms—7	No difference	37.0 ± 12.3 pg/mL	38.2 ± 12.8 pg/mL	−	Atack, 1988 [62]
CSF	CTL—40; AD—36; VAD—26	No difference	144.5 ± 10.9 pmol/L	138.3 ± 6.2 pmol/L	−	Heilig, 1995 [63]
Plasma	CTL—25; AD—25	Decreased compared to CTL	143 ± 21 fmol/mL	254 ± 29 fmol/mL	Not examined	Koide, 1995 [65]
Plasma	CTL—14; AD—14	No difference	48.5 + 10.43 pmol/L	41.5 + 13.26 pmol/L	Not examined	Proto, 2006 [66]

**Table 2:** Neuroprotective properties of NPY.

Material	Model	NPY Administration	Effects of NPY Administration	Reference
Primary neurons treated with Aβ 1–42.	In vitro	24 h pre-incubation with amidated C-terminal fragments of NPY 21–36 and 31–36 (dilutions ranging from 1 nm to 10 μm).	Protected human neuronal cultures from the neurotoxic effects of Aβ	Rose et al., 2009 [73]
SH-SY5Y human neuroblastoma cells treated with Aβ 25–35 peptide	In vitro	24 h pre-incubation with different NPY concentrations (0.5, 1 and 2 μM)	Prevented cell death.Restored levels of NGF (nerve growth factor) and BDNF protein and mRNA.	Croce et al., 2011 [75]
Primary neurons treated with Aβ 1–42.	In vitro	24 h pre-incubation with different NPY concentrations (0.5, 1, and 2 μM)	Increased cell viability. Increased NGF synthesis, normalized NGF release, and downregulated NGF mRNA expression.	Croce et al., 2012 [76]
Primary rat cortical neurons treated with Aβ	In vitro	24 h pre-incubation with NPY (1 μM)	Prevented cell death, increased level of mRNA BDNF	Croce et al., 2013 [77]
B103 rat neuroblastoma cells or adult rat hippocampal neuronal progenitor cells treated with Aβ	In vitro	24 h pre-incubation with a lentiviral vector expressing NPY with a blood–brain barrier transport tag (ApoB)	Increased neuronal survival	Spencer et al., 2016 [78]
Transgenic mice expressing high levels of human neprilysin and APP	In vivo	i.c.v. administration of amidated C-terminal fragments of NPY 21–36 and 31–36 (for 28 days)	Decreased the area occupied by MAP2-immunoreactive dendrites in the neocortex. Ameliorated the neurodegenerative pathology	Rose et al., 2009 [73]
Mice after intravenous injection of Aβ 1–40	In vivo	Single i.c.v. administration of NPY (0.0234 mol/L) 15 min before i.c.v. infusion Aβ administration	Reduced oxidative stress in the prefrontal cortex and hippocampus.	dos Santos et al., 2013 [74]
Mice expressing human APP751 (Line 41) under control of the mThy-1 promoter (APP-tg)	In vivo	A single intraperitoneal injection of Lentiviruses expressing the NPY-apoB (1 × 10^9^ transducing units in a volume of 300 μL) with a blood–brain barrier transport tag	The NPY-apoB did not reduce Aβ accumulation, but increased neurogenesis and synaptic density, and resulted in behavioral improvements and widespread reduction in astrogliosis.	Spencer et al., 2016 [77]

## Abbreviations

AD	Alzheimer’s disease
APP	amyloid precursor protein
Aβ	amyloid beta
BDNF	brain-derived neurotrophic factor
CNS	central nervous system
CSF	cerebrospinal fluid
FTD	frontotemporal dementia
NGF	nerve growth factor
NPY	neuropeptide Y
